# Neurophysiologic Characteristics of Motoric Cognitive Risk Syndrome: A Multimodal Assessment of EEG, Physical and Cognitive Function

**DOI:** 10.1002/alz70858_099557

**Published:** 2025-12-25

**Authors:** Eunseon Noh, Hye‐Jin Park, Seongryu Bae, Jiwon Yang, Jaewon Choi, Kai Wang, Jong‐Hwan Park, Hyuntae Park

**Affiliations:** ^1^ Department of Health Sciences, The Graduate School, Dong‐A University, Busan, Korea, Republic of (South); ^2^ Digital Healthcare Institute, Dong‐A University, Busan, Korea, Republic of (South); ^3^ Pusan National University Hospital, Busan, Korea, Republic of (South)

## Abstract

**Background:**

Motoric cognitive risk syndrome (MCR) is a pre‐dementia syndrome defined by slow gait speed combined with subjective cognitive function decline, is a powerful clinical tool used to identify older adults at a high risk of developing dementia. This investigation aimed to uncover the distinctive neurophysiological characteristics of MCI and MCR through a comprehensive assessment.

**Method:**

Eight‐seven community‐dwelling older adults (aged 65‐85 years) were classified into MCR (*n* = 27), mild cognitive impairment (MCI; *n* = 16), and healthy control (HC; *n* = 44) groups. Participants underwent physical function measurements, including gait analysis and functional mobility tests (timed up and go, five times sit‐to‐stand test, etc.). Cognitive function (mini‐mental state examination, single digit span test, etc.) was also assessed. Furthermore, brain function was assessed using resting‐state EEG with a 21‐channel headset.

**Result:**

Compared to the HC, both MCI and MCR groups showed significantly lower physical and cognitive functions overall (*p* < .001). MCR group exhibited significantly lower performance in gait speed, functional mobility, and executive function compared to the MCI group (*p* < 0.05). EEG analysis revealed that both MCI and MCR groups showed significant decrease in theta power in specific regions compared to the control group (*p* < 0.05). MCR group demonstrated higher theta/beta ratios compared to the MCI and HC group, with a significant progressive increase observed across the groups (HC < MCI < MCR, *p* < 0.05).

**Conclusion:**

This study examined the neurophysiological characteristics of MCR through a comprehensive analysis of motor‐cognitive integration, examining differences in physical, cognitive, and neurological functions across the three groups: HC, MCI, and MCR. In particular, EEG analyses revealed distinct neurophysiological signatures unique to MCR. These neurophysiological markers represent a promising community‐based screening tool enabling the rapid identification of MCR in older populations and facilitating the early detection of high‐risk individuals for timely and appropriate management.

